# Getting by on credit: how district health managers in Ghana cope with the untimely release of funds

**DOI:** 10.1186/1472-6963-6-105

**Published:** 2006-08-17

**Authors:** Augustine D Asante, Anthony B Zwi, Maria T Ho

**Affiliations:** 1 National Centre in HIV Social Research, The University of New South Wales, Sydney, Australia; 2 School of Public Health and Community Medicine, The University of New South Wales, Sydney, Australia

## Abstract

**Background:**

District health systems in Africa depend largely on public funding. In many countries, not only are these funds insufficient, but they are also released in an untimely fashion, thereby creating serious cash flow problems for district health managers. This paper examines how the untimely release of public sector health funds in Ghana affects district health activities and the way district managers cope with the situation.

**Methods:**

A qualitative approach using semi-structured interviews was adopted. Two regions (Northern and Ashanti) covering the northern and southern sectors of Ghana were strategically selected. Sixteen managers (eight directors of health services and eight district health accountants) were interviewed between 2003/2004. Data generated were analysed for themes and patterns.

**Results:**

The results showed that untimely release of funds disrupts the implementation of health activities and demoralises district health staff. However, based on their prior knowledge of when funds are likely to be released, district health managers adopt a range of informal mechanisms to cope with the situation. These include obtaining supplies on credit, borrowing cash internally, pre-purchasing materials, and conserving part of the fourth quarter donor-pooled funds for the first quarter of the next year. While these informal mechanisms have kept the district health system in Ghana running in the face of persistent delays in funding, some of them are open to abuse and could be a potential source of corruption in the health system.

**Conclusion:**

Official recognition of some of these informal managerial strategies will contribute to eliminating potential risks of corruption in the Ghanaian health system and also serve as an acknowledgement of the efforts being made by local managers to keep the district health system functioning in the face of budgetary constraints and funding delays. It may boost the confidence of the managers and even enhance service delivery.

## Background

District health systems, comprising of primary health care and first referral hospitals, remain pivotal in the organisation and delivery of basic health services in developing countries [1,2]. However, their effectiveness in many settings has been constrained by poor planning, inefficient resource management, staff shortages and inadequate funding. Since the emergence of the Primary Health Care (PHC) concept in 1978, emphasis has been placed on strengthening district health systems to enhance their capacity to deliver services more effectively. Decentralisation has been one of the key policy reforms implemented to strengthen district health management and planning capacities. Many developing countries have in the past two decades implemented health sector decentralisation which has resulted in substantial transfer of planning and managerial responsibilities to district health managers [3,4].

However, while decentralisation has empowered district managers and granted them more control over budgetary resources, the allocation of funds from the central government in many countries has not been streamlined to support the decentralised system. In sub-Saharan Africa, district health systems depend largely on government grants, which have traditionally been inadequate for the services for which they are allocated. In general, Africa has the lowest per capita health spending in the world, ranging from less than US$20 in the poorest countries such as Sierra Leone and Liberia to about US$50 in the more affluent ones like South Africa and Botswana [5]. Only few countries on the continent meet the US$34 per capita spending estimated by the WHO Commission on Microeconomics and Health as needed for the delivery of a set of essential interventions [6].

To increase financial resources for health, many African governments have introduced reform mechanisms including health insurance schemes and user fees. These reforms have had varying degrees of success. However, the pressure of dealing with the chronic resource scarcity largely remains and has diverted the attention of governments from effective allocation of available resources to resource mobilisation [7,8]. To date, many African countries do not have any established systems for efficient and equitable allocation of public sector health funds across populations and jurisdictions as well as between tertiary and primary health care. Often, the allocation of government funds are historically based and tends to favour tertiary health care as opposed to primary health care as well as benefiting the rich more than the poor [9,10]. Another chronic problem is the delay in the allocation of health funds from the Ministry of Health to lower levels of the health system. While timely allocation of funds is crucial for supporting decentralised health systems [8], regional and district health authorities in Africa often have to endure long delays in funding which weakens further their already precarious financial positions. The aim of this paper is to examine how the untimely release of public sector health funds in Ghana affects district health activities and the way district managers cope with the situation.

### Overview of funding of district health systems in Ghana

Ghana is among the poorest countries in the world. It is ranked 138 out of 177 countries on the UNDP Human Development Index for 2005 [11]. Nearly 40% of its estimated 20.4 million population live in poverty; about 27% survive on a dollar or less per day. Politically, it has been a relatively stable country in the turbulent region of West Africa. It has a functioning multi-party democracy and has manifest economic growth in recent years. Like many countries in sub-Saharan Africa, Ghana faces serious health challenges. Non-communicable diseases such as diabetes and cerebrovascular disease are on the rise [12,13] but the leading causes of death remain poverty-related diseases, notably malaria. Table 1 presents selected health-related indicators for Ghana, Zambia and Malaysia (see appendix for reasons for comparing Ghana with Zambia and Malaysia).

The government, in conjunction with donors, has been making efforts to improve the health status of the population over the past decade. Major challenges are present in relation to maximising funding and the appropriate allocation of funds. Although the percentage of total government expenditure on health increased from 5.7% in 2000 to 7% in 2004 [14], in per capita terms, health spending in Ghana remains one of the lowest among developing countries. The per capita total expenditure on health in 2003 (at average exchange rate) was only US$16 compared to that of US$28 in neighbouring Côte d'Ivoire [15]. The allocation of funds within the health system has largely been historically based. The pattern of allocation of the previous year is used as a guide for the current year's allocation. It has little bearing with the health needs of the population. Despite policy commitment to enhance the resource allocation formula to incorporate health needs, poverty and gender issues, only minor changes have been made [16,17].

Much recently, there have been serious moves towards pro-poor and needs-based resource allocation in the health sector. The MOH ring-fences part of the health budget and lodges it with specific Budget and Management Centres (BMCs) to protect critical service areas from the risk of under-funding. BMCs are cost centres created at all levels of the Ghanaian health system to independently manage funds. Their financial management capacity are thoroughly assessed and approved before they are given the nod to hold and manage funds [17]. The ring-fencing is also used to ensure that resource allocation patterns reflect national poverty alleviation and equity commitments. Further to this, the government allocates a significant portion (6.47% as of 2003) of its debt relief funds under the World Bank/IMF sponsored Highly Indebted Poor Country (HIPC) initiative to support pro-poor health activities. These include payment of exemptions for maternal deliveries and Guinea Worm eradication in northern Ghana [18]. Notwithstanding these efforts, resource allocation in the Ghanaian health sector remains inadequately reflected on the health needs of the population, and to date, only few indicators of need are included in the allocation criteria.

The 1995 Medium Term Health Strategy (MTHS) and the subsequent Sector Wide Approach (SWAp) which seeks to create a common basket for funding MOH priorities [19], tackle some of the systemic problems in the health system. One area of emphasis has been the strengthening of the district health system including the financial management system [20]. The financial management reform that followed SWAp shifted management responsibilities to the district level and granted greater control over funds to local managers. District Health Administrations (DHAs) in the country have, since 1999, been receiving and directly managing funds for non-salary recurrent expenditure [21] under the BMC concept. While this has been hailed as a boost to district health delivery, release of funds to districts has remained untimely and unpredictable. First quarter allocations expected in January are often received in the second quarter, sometimes as late as June. Fourth quarter allocations may not be received at all. This erratic flow of funds to district health services threatens to offset any potential benefits from the reforms. This sub-study is one component of a broader research project on resource allocation and equity in the Ghanaian health sector. Findings of other components have been reported elsewhere [22,23].

## Methods

### *Setting and participants*

**Figure 1:**
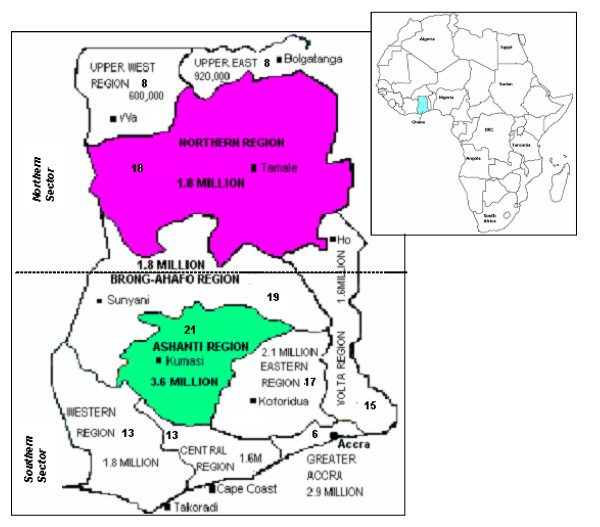
A map of Ghana showing the ten administrative regions, total population and the number of districts in each region.

Ghana is divided into ten administrative regions, each of which is sub-divided into a number of districts. There are a total of 138 districts in the country. The Ashanti region is the most populous with over 3.6 million inhabitants living in 21 districts. Northern region is the biggest in terms of landmass covering about 70,384 km^2^. It has a population of about 1.8 million and 18 districts (figure 1).

Population of districts ranges from as low as about 52,000 in the Kadjebi District to as high as 1.7 million in the Accra Metropolitan District. For the purposes of this study we selected two of the ten regions: Northern and Ashanti, covering the northern and southern sectors of the country. Justifications for the selection of the two regions have been outlined in Asante *et al*. 2006 [22].

Four districts from each region were strategically selected on the basis of population size and geographical location (whether rural or urban). These were the Tamale Municipal, Savelugu-Nanton, West Gonja and West Mamprusi districts from the Northern region, and the Kumasi Metropolitan, Sekyere-East, Amansie East and Asante-Akim North from the Ashanti region. We interviewed 16 purposively selected district managers, comprising the eight directors of health services and the eight district health accountants. Seven of the directors were trained physicians with additional responsibilities of attending to patients at the district hospitals. Each district director of health services was given a letter of introduction from The University of New South Wales (School of Public Health) explaining the purpose of the study and seeking their cooperation. The participants were verbally asked if their interviews could be tape-recorded. None of them raised any concerns about participating in the study or being recorded. To ensure confidentiality of information and protect the identity of participants, only the researchers were involved in the transcription, analysis and presentation of data. The analysis and presentation of data did not enable identification of individual participants.

### *Data collection*

Semi-structured interviews were conducted with the 16 participants between September 2003 and February 2004. The interviews covered when and how funds are channelled to districts from the national level, processes of accessing released funds; the differences between donor and government funds in terms of size, flows and conditions of use; the effects of funding flows on implementation of programmes and how managers cope with delays in fund releases. We triangulated data from documentary sources and interviews at national and regional levels and with other stakeholders including donors to validate the information gathered from the district managers. Evidence of precisely when funds were transferred from national and regional levels to districts, the amount of money involved, and when district bank accounts were credited was gathered from administrative records to validate managers' accounts of funding delays and inadequacy. One of the authors (ADA) conducted all the interviews, each of which was audiotaped and lasted for about an hour.

### *Analysis*

Interview tapes were transcribed verbatim and critically examined using content analysis [24]. The data from all districts were combined, given that there were no major differences between regions in respect of the data reported on this study. Transcripts were repeatedly read before and after coding to ensure proper categorisation of data. Categories were initially developed in line with the main domains covered in the interviews but this changed following a more detailed reading of information under each category to identify emergent themes and sub-themes. We carefully examined and discussed data that did not fit well any of the categories developed to ensure that their exclusion from the study would not negatively affect the results.

## Results

### How untimely is the release of funds?

Apart from salaries that were generally paid on time, there were delays with the release of other line items. All 16 respondents mentioned that quarterly transfers for non-salary recurrent expenditures (i.e., government funds for administration and service expenditures) from the national level had been inadequate and unpredictable in recent years. Historical data obtained for 2002 showed an extremely low allocation from government sources to all districts. Table 2 presents the amount of funds transferred to the eight districts in this study for 2002 in respect of service expenditure. The level of government transfers to districts (excluding funds for sub-districts) was extremely low; all of the eight DHAs combined received less than US$25,000 for the whole year. In per capita terms, all districts received less than US$5 per 100 people.

Funds for non-salary recurrent expenditures are released on a quarterly basis. Government funds for service expenditures are released in the form of a cheque. The cheques are channelled to DHAs through the regional health administration (RHA). The same procedure is used for the release of donor-pooled funds (DPF), which can be used for all expenditures except salaries. Government funds for administrative expenditure are released in a form of *expenditure warrants *through the District Assembly (DA). Salaries are paid through the banking system.

Managers were highly critical of the size and unpredictable flow of funds, as well as the cumbersome procedures for accessing the government administration funds. One district director of health services made the following observation about the untimely releases of funds for administrative expenditure:

"The timing is bad; I mean bad, really bad! They are not regular at all…we understand that the budget has to go through some process, say from the district to the region, to national, then it will go to Ministry of Finance and probably Parliament has to approve it before the money can be released. But that is no excuse, something has to be done about it; they should find a way to solve it. Sometimes you stay up to June and nothing has come, meanwhile that is half the year gone so what services are you going to render and with what? Sometimes it is so demoralising you just don't know what to do".

Critical comments about the flow of government funds were common across the districts. Evidence from documents and interviews at regional and national levels confirmed that there had been delays in releasing funds, particularly government funds for administrative expenditure. At the time the interviews were conducted (December 2003), many districts were still seeking to obtain their third quarter allocations for administrative expenditure and most respondents doubted whether fourth quarter allocations would be released before the year ends. With the exception of one person who said funding flows from all sources have been similarly unpredictable, all other respondents expressed relative satisfaction with the release and flow of the donor-pooled funds.

### How does the untimely release of funds affect district health care delivery?

The untimely release of funding had two major effects on health care delivery. These were the inability to implement planned programmes on time, mentioned by all respondents, and the negative impact on staff morale.

"Funding from government doesn't come in time. It throws all our plans in disarray. We do what we have planned to do in the first quarter mostly in the second quarter and the second quarter in the third quarter. As for fourth quarter activities, if we wait for government funds we won't do it because the funds are normally not received".

Postponing implementation of programmes as a result of delays in funding happened regularly in all districts. One respondent recounted a situation where there was an outbreak of cerebrospinal meningitis (CSM) in his district and the district director was "frantically running up and down looking for money to control the disease" while their allocation for the quarter was "locked up in Accra". The level of frustration that district managers face over insufficient allocations and the difficulty of accessing government funds for administrative expenditure was echoed in the account of one district health accountant who spent a week in the capital (Accra) chasing their first quarter allocation.

"You spend a lot to chase the money, meanwhile our total allocation for the first quarter last year was just a little over 2 million cedis, I spent one week in Accra chasing it…By the time we received it nearly one million, about half of the money is gone. So that is the problem we face – very frustrating, very, very frustrating! At times you feel like just leaving the money and saving yourself the trouble of making all those desperate chase for that peanut".

The majority of the respondents reported that the delays in receiving funds from the government seriously affected staff morale. A manager in the Northern region observed that because they have no access to telephones "sometimes personnel from remote sub-districts would travel all the way to the DHA for their allocations, only to be told that the money is still not received". He queried whether anything "can be more frustrating than this".

### How do managers cope with the untimely releases?

#### *Obtaining supplies on credit*

All managers reported obtaining supplies on credit and paying for them when funds are received. Since the DHA deals more with public health issues such as immunisation, health education and disease eradication, outreach programmes are the core of their activities. This has made fuel one of the most important items of expenditure. All 16 interviewees reported that in order to continue operating, they obtain fuel and other supplies on credit and pay for them when funds eventually arrive.

"The issue is that because the situation is not new, our suppliers have come to understand it and so they supply us things on credit and when the money comes we pay them. It's a big problem because it is not easy to be crediting things all the time; there is always a limit to what somebody can supply to you on credit".

#### *Borrowing cash internally*

Apart from obtaining supplies on credit, there were reports of internal cash borrowings from the district hospitals' internally generated fund (IGF). DHAs in Ghana generally do not themselves generate funds internally. However, the sub-district facilities and the district hospitals are permitted to retain any IGF revenue they generate. Three district directors and their accountants stated that occasionally the DHAs borrow money from the district hospital in order to continue with their activities.

"The district hospitals have their own problems, but at least, they have some IGF to fall on when things delay. Since we are here together, they understand the difficulties we face, so we borrow from them from-time-to-time and pay back when we get our money".

This practice appeared widespread in districts where the directors of health services were also the only or key medical officers at the district hospital.

#### *Pre-purchasing and saving of materials*

Based on past experience of delays in funding, particularly in the first quarter, district managers often try to lessen the effects of the erratic funding flows on their activities by taking "pre-emptive" action. Over two-thirds of the respondents mentioned that government accounting regulations do not allow them to carry forward to the following year, any balances in their accounts for government funds. Such balances are automatically forfeited after 31 December; the end of the financial year. Consequently, whenever there are any cash balances on government funds in December, they used it to purchase some of the materials needed in the first quarter to avoid forgoing such funds. Even though in principle, this is against government regulations, in practice, managers see it as necessary to keep the health system running in the first quarter.

#### *Conserving donor funds for first quarter*

Many studies have noted the crucial role of donor funding in supporting health delivery in Africa [25]. In Ghana, donor funding constitutes about 35% of the total health budget [16,17]. The majority of district managers confirmed that donor-pooled funds (DPF) have been the most reliable source of funding for district health care delivery. They spoke enthusiastically about how donor funds help them to partially operate in the first quarter. A manager in the Northern region provided the following contrast between the DPF and government funds:*"I can tell you that without DPF the health system would have collapsed long ago. Government funds are too small and very unreliable. You never know when they are coming and cannot be sure how much you are getting*". In coping with the delays, more than half of the respondents revealed that they do their best to conserve some of their donor funds for the fourth quarter for use in the first quarter:

"We depend mostly on our DPF balance for the first and parts of the second quarter...thus, if we have balance brought forward from the previous year. But we try our best to, at least, save something from our fourth quarter DPF allocation otherwise we'll be in big trouble".

## Discussion

The problems confronting health systems in sub-Saharan Africa are well documented [26]. What is less known are the strategies that health workers in different settings adopt to cope with the challenges they face in their day-to-day delivery of services. Knowledge of such informal coping mechanisms offers insights into local management practices which can be adapted and introduced, and others which are less acceptable.

This study suggests that the untimely release of funds, particularly in the first quarter, is a significant barrier to effective health service delivery in many districts. In the Northern region, where the road network is largely underdeveloped, it is within the first quarter that most isolated communities can conveniently be reached, as the dry weather permits the use of motorcycles and bicycles on the narrow bush-paths to these communities. However, the first quarter is the period in which funds are often not received. The delays have forced managers to resort to several unconventional practices such as obtaining supplies on credit, borrowing internally from other parts of the health service, pre-purchasing materials, and conserving donor funds, to keep the health system running.

Obtaining supplies on credit is the most common coping mechanism. Managers have formed informal alliances with suppliers who are willing to supply on credit and be paid later. To a large extent, it resembles a public-private-partnership between the District Health Administrations – the local agencies representing the central government, and suppliers of the private sector. However, public-private partnerships are formal mechanisms often with official government approval and encouragement. This is unofficial and occurs without the approval of the central government. The practice, nonetheless, provides managers with fuel and other vital supplies in the absence of funds and appears to have contributed significantly towards maintaining service delivery at the district level in Ghana. It entails, however, a potential risk of corruption. Unscrupulous managers can easily team up with suppliers and over-invoice the DHA for "ghost" supplies. Government involvement to formalise this strategy, for example, through the District Assemblies, may reduce the risk of corruption and give confidence to both suppliers and managers.

Pre-purchasing materials towards the end of the year with balances on government funds against the first quarter is another common strategy used to cope with delays in funds availability. This practice was a direct response to the government's accounting policy that forbade the carrying forward of balances on government funds to the following year. Although funds for district health delivery are insufficient, the late releases means that balances may be underutilised. In order not to forfeit such balances, managers spend them on purchasing materials against the first quarter before 31 December. This could, again, lead to inefficient and irrational use of funds, as there is pressure on managers to either spend or forfeit their closing balances. A change in policy to allow balances to be rolled over to the following year would seem sensible.

Finally, in contrast with the pressure to spend government balances before 31 December, managers try to conserve part of their DPF and carry it forward to the following year in anticipation of delays in the first quarter. This is possible because donors allow the closing balances on the 'pooled' funds to be rolled over to the following year. Most managers expressed satisfaction with this arrangement and the general flexibility associated with the use of DPF. One accountant commented: "the flexibility reduces the pressure on us to spend". Indirectly, this may promote prudent use of limited resources.

### Implications for equity and the National Health Insurance Scheme

The findings of this study, as discussed above, have several implications for equity and the National Health Insurance Scheme (NHIS) currently being implemented in Ghana. The NHIS was introduced to increase funding to the health sector, reduce financial barriers to accessing services and ensure equitable access especially for the poor [18]. For these goals to be achieved, prompt release of funds to scheme managers from the government is essential, in particular, funds to cover the premium of the very poor and vulnerable populations who cannot afford payment of the requisite premiums. If the flow of funds from the national level to districts continues to be as erratic as it currently is, it will lead to scheme managers inevitably denying those poor and vulnerable consumers whose premiums are covered by the government, access to treatment, thereby defeating the scheme's key objective of equitable access.

Similarly, under the current NHIS proposal the government would engage in some sort of 'risk equalisation' by providing subsidies to make up for the variations in income generated from insurance premiums in different areas. Such subsidies will be disbursed from a centrally- administered tax and donor financed National Health Insurance Fund (NHIF). If the current bottlenecks in disbursement of funds from the national level are not rectify, this may affect the allocation of subsidies from the NHIF to equalise the amount of premium generated across schemes in different parts of the country, thereby perpetuating the existing geographical inequities in funding of health activities.

### Limitations of the study and suggestions for further work

The selection of two regions and four districts from each region limits the generalisability of the findings. Although these regions and districts were strategically selected to provide representative insights into what is happening throughout the country, a much broader picture of how delays in funding impact on health care delivery and the way managers cope with the situation could have been formed if more regions and districts had been covered. In the light of this, there is the need for further work into how managers cope with funding delays in other regions and districts so that a more comprehensive picture of the situation could be formed and concrete nation-wide policy directions prescribed.

There have also been recent developments in the health sector with key donors opting to provide budgetary support under the multi-donor budget support (MDBS) initiative rather than sector specific support as was the case when this study was conducted. Although not a limitation of the current study as such, this move could potentially change the pattern and pace of resource allocation in the health sector. A further research to assess how this change in direction by donors has affected resource flow would be a useful project.

## Conclusion

The untimely release of health funds coupled with the low levels of funding undermines district health delivery in Ghana. Among other things, it undermines the morale of health professionals and impedes the implementation of planned activities. District managers do their best to cope with the situation by developing several informal strategies to 'get by'. This demonstrates the commitment of these managers to keep the district health system running despite difficulties. However, some of the coping mechanisms are open to abuse and need to be closely examined. Government involvement to formalise some of the more effective strategies such as the crediting of supplies, would not only reduce the potential risk of corruption, but could also boost the confidence of district managers and enhance service delivery. While poor funding of health systems in Africa is a major development problem, the untimely release of funds is an administrative bottleneck that can and must be overcome. An effective resource allocation system which allows central government grants for district health services to be released on time at the beginning of every quarter will make a difference in the way health care is delivered at the district level in Ghana.

## Competing interests

The author(s) declare that they have no competing interests.

## Authors' contributions

ADA planned the study, collected and analysed the data, and prepared the first draft of this manuscript. ABZ and MTH contributed to the design of the study, oversaw the collection and analysis of data, and commented on the first and subsequent drafts of this manuscript. The data were collected for ADA's PhD thesis which was supervised by ABZ and co-supervised by MTH. All authors read and approved the final manuscript.

## Appendix

The basis for comparing Ghana with Zambia and Malaysia was one of pragmatism than scientific. Zambia was of interest mainly because the model of health sector reforms pursued in that country under the sector-wide approach (SWAp), including financial management reforms and decentralisation was similar to those undertaken in Ghana. These similarities have long been a source of interest to researchers and attracted several comparative studies including Cassels and Janovsky (1996) and Bossert and Beauvais (2002). The interest in Malaysia stems from its history ties with Britain; like Ghana, Malaysia was a British colony which became independent in 1957 – the same year that Ghana also gained independence from Britain. The economic fortunes of the two countries at the time of independence were similar, providing a reasonable basis for comparison.

## Pre-publication history

The pre-publication history for this paper can be accessed here:



## Figures and Tables

**Table 1:** Selected health-related indicators

**Indicators**	**Ghana**	**Zambia**	**Malaysia**
Population (millions 2003)	20.4	10.4	24.8
GNI per capita (US$ 2003)	320	380	3,780
Life expectancy (2002)	55	37	73
Under-5 mortality/1000 (2002)	97	182	8
Adult literacy (% 15 and above- 2002)	74	80	89
Malaria cases/100000 (2000)^b^	15,344	34,204	57
TB cases/100 000 (2003)^b^	369	508	135
HIV prevalence rate (% 2003)^b^	3.1	16.5	0.4
Government Health spending % GDP (2002)^b^	2.3	3.1	2.0
Health spending per capita (PPP US$ 2002)^b^	73	51	349
Physicians/100,000 people (1990–2004)^b^	9	7	70

**Source**: World Development Report 2005.

^b ^Human Development Report 2005

**Table 2:** Districts by government allocation for service expenditure (excl. donor funds) – 2002

**District (DHA only)**	**Service Expenditure (Cedis)**	Service Expenditure (US$)*	**Service Expenditure per 100 people (US$)**
***Northern***			
Tamale municipal	39,019, 000	4,758.41	1.62
West Gonja	41,930, 000	5,113.41	3.67
West Mamprusi	38,001,000	4,634.26	4.03
Savelugu-Nanton	29,427,000	3,588.65	3.99
***Ashanti***			
Kumasi Metro	14,952, 607	1,823.49	0.16
Amansie East	14,156, 921	1,726.45	0.77
Sekyere East	14,096,000	1,719.02	1.09
Asante-Akim North	12,162,716	1,483.26	1.17
**Total**	**203,745,244**	**24,846.95**	

**Source**: Northern Regional Health Administration- *Analysis of Recorded REPAC Data. GoG Reconciliation Report for Subvention Accounts *December 2002.

Ashanti Regional Health Administration- *Global Expenditure Report*, December 2002

*Exchange rate – ¢8200 to US$1
